# Patients phenotypes and cardiovascular risk in type 2 diabetes: the Jackson Heart Study

**DOI:** 10.1186/s12933-022-01501-z

**Published:** 2022-06-01

**Authors:** Justin B. Echouffo-Tcheugui, Solomon K. Musani, Alain G. Bertoni, Adolfo Correa, Ervin R. Fox, Robert J. Mentz

**Affiliations:** 1 Department of Medicine, Division of Endocrinology, Diabetes and Metabolism, Johns Hopkins University School of Medicine, 5510 Bayview Circle, Baltimore, MD 21224 USA; 2 Department of Medicine, Jackson Heart Study, University of Mississippi Medical Center, Jackson, USA; 3 Department of Epidemiology and Prevention, Wake Forest University School of Medicine, Winston-Salem, NC USA; 4 Department of Medicine, Division of Cardiology, University of Mississippi Medical Center, Jackson, USA; 5 Department of Medicine, Division of Cardiology, Duke University School of Medicine, Durham, NC USA

**Keywords:** Diabetes, Cluster analysis, Comorbidities, Echocardiography, Biomarkers, Outcomes

## Abstract

**Background:**

Cardiovascular prognosis related to type 2 diabetes may not be adequately captured by information on comorbid conditions such as obesity and hypertension. To inform the cardiovascular prognosis among diabetic individuals, we conducted phenotyping using a clustering approach based on clinical data, echocardiographic indices and biomarkers.

**Methods:**

We performed a cluster analysis on clinical, biochemical and echocardiographic variables from 529 Blacks with diabetes in the Jackson Heart Study. An association between identified clusters and major adverse cardiovascular events (MACE- composite of coronary heart disease, stroke, heart failure and atrial fibrillation) was assessed using Cox proportional hazards modeling.

**Results:**

Cluster analysis separated individuals with diabetes (68% women, mean age 60 ± 10 years) into three distinct clusters (Clusters 1,2 &3 - with Cluster 3 being a hypertrophic cluster characterized by highest LV mass, levels of brain natriuretic peptide [BNP] and high-sensitivity cardiac troponin-I [hs-cTnI]). After a median 12.1 years, there were 141 cardiovascular events. Compared to Cluster1, Clusters 3 had an increased risk of cardiovascular disease (hazard ratio [HR] 1.60; 95% confidence interval [CI] 1.08, 2.37), while Cluster 2 had a similar risk of outcome (HR 1.11; 95% CI 0.73, 168).

**Conclusions:**

Among Blacks with diabetes, cluster analysis identified three distinct echocardiographic and biomarkers phenotypes, with cluster 3 (high LV mass, high cardiac biomarkers) associated with worse outcomes, thus highlighting the prognostic value of subclinical myocardial dysfunction.

**Supplementary Information:**

The online version contains supplementary material available at 10.1186/s12933-022-01501-z.

## Introduction

Cardiovascular disease and type 2 diabetes (diabetes) are common and co-occurring conditions [1, 2]. Individuals with diabetes are at increased risk of cardiovascular diseases, including coronary artery disease [3], stroke [4], heart failure [5], and atrial fibrillation [6]. Contemporary clinical trials of diabetes medications, namely sodium-glucose co-transporter-2 (SGLT-2) inhibitors [7] and glucagon-like peptide 1 (GLP-1) receptors agonists [8], have shown significant cardiovascular benefits among individuals with diabetes. The results of these trials are corroborated by studies of the potential effects of SGLT2 inhibitors on pathways linking diabetes to heart failure, including insulin resistance, myocardial fat accumulation, cardiac function, cardiac metabolism, as well as arterial stiffness [9, 10]. The results of the SGTL2 inhibitors and GLP-1 receptors agonists trials have made it an imperative to refine our understanding of the cardiovascular risk among individuals with diabetes, as this will guide an appropriate implementation of these novel therapies.

Diabetes tends to track with other cardiometabolic conditions, thus any assessment of diabetes-related myocardial dysfunction and its prognostic value should account for the comorbidities. Indeed, diabetes often coexist with comorbidities such as obesity and hypertension [11]. Obesity [12–14], and hypertension [13, 14], may also lead to myocardial alterations, which may bear some similarities to the diabetes-related myocardial changes. Thus, the specific individual and synergistic contributions of various causative factors to diabetes-related cardiac dysfunction is unclear. Cluster analysis, a hypothesis-free approach (as opposed to classic statistical analyses) to risk estimation [15, 16], may allow a refined phenotyping, and thus provide novel insights into contribution of various risk factors to the heightened cardiovascular risk among patients with diabetes.

We used data from the community-based Jackson Heart Study comprised of black adults to identify clusters of cardiac phenotypes among individuals with diabetes. We also examined the distribution of clinical and echocardiographic parameters that may better define cardiovascular prognosis.

## Methods

The Jackson Heart Study recruited 5306 Blacks (African Americans), aged 21 to 94 years, from the Jackson, Mississippi, metropolitan area [17]. The Jackson Heart Study design and methods have been described elsewhere [17]. The present study included participants who attended examination 1 (2000–2004), underwent an echocardiography and were found to have diabetes (n = 1123). The diabetes status was defined using the American Diabetes Association criteria as a fasting plasma glucose ≥ (126 mg/dL) or HbA_1C_ ≥ 6.5% [18], self-reported diabetes or confirmed use glucose lowering medications, or a self-report of physician-diagnosed diabetes. As shown in Additional file 1: Fig. S1, we excluded participants with a history of cardiovascular disease (including history of coronary artery disease, cardiomyopathy/heart failure including valvular heart disease [moderate or greater mitral regurgitation and aortic insufficiency] and the presence of regional LV wall motion abnormalities), missing data on echocardiographic variables, and missing data on other variables including brain natriuretic peptide (BNP), and high-sensitivity cardiac troponin-I (hs-cTnI) and other variables. After applying exclusions, the final analytic sample was 529 adults. The comparison of individuals that were included in the study to those excluded is shown in Additional file 1: Table S1.

 The study protocol was approved by the institutional review board of the University of Mississippi Medical Center, Jackson State University and Tugaloo College. All the participants provided informed consent.

The cardiac ultrasound examinations were undertaken using a Sonos 4500 cardiac ultrasound machine (Hewlitt Packard, Andover, MA). Measurements, including two-dimensional and Doppler flow assessments, were performed offline by a trained echocardiographer based on American Society of Echocardiography recommendations [19]. Left ventricular End-Diastolic Volume (LVEDV) and LV End-Systolic volume (LVESV) were indexed to body surface area, and LV mass was measured in M-mode and was calculated using the American Society of Echocardiography–corrected formula: LV mass (g) = 0.8 × 1.04 [(LV end diastolic diameter + IVST + PWT)^3^– (LV end diastolic diameter)^3^] + 0.6, where IVST is the interventricular septal wall thickness and PWT is the posterior wall thickness. For this analysis, LV hypertrophy was defined as an LV mass indexed to body surface area (BSA) as per the American Society of Echocardiography (ASE) criteria > 95 g/m^2^ for women and > 115 g/m^2^ for women [20], and a low ejection fraction was defined as an LV ejection fraction < 50%. Using pulsed wave Doppler, mitral inflow velocities and peak early (E) and late (A) diastolic velocities were measured, and E/A ratio was calculated [21].

Clinical information including demographic characteristics, medical history and medication use, were assessed by standardized questionnaires, physical examination, and laboratory tests. The methods of risk factor ascertainment in the Jackson Heart Study have been reported elsewhere [17]. Current smokers were defined as those who reported having smoked ≥ 1 cigarette per day regularly during the year preceding the examination. Height, and weight were measured and body mass index was calculated (kg/m^2^). Blood pressure was measured twice in the left arm of the seated subject with a mercury column sphygmomanometer. The average of the two readings was used as the examination BP, and hypertension was defined as systolic blood pressure ≥ 140 mmHg or diastolic blood pressure ≥ 90 mmHg, or self-reported antihypertensive medication use. Serum creatinine was measured using the rate Jaffe reaction, and the kidney function was assessed using the estimated glomerular filtration rate calculated by the Chronic-Kidney Disease—EPI study equation [22].

Plasma total cholesterol, high-density lipoprotein (HDL) cholesterol, and triglycerides concentrations were measured using standard enzymatic methods, on a Vitros 950 or 250, Ortho‐Clinical Diagnostics analyzer (Raritan, NJ) in accordance with the College of American Pathologists Proficiency Testing Program [23]. Low-density lipoprotein (LDL) cholesterol was calculated using the Friedewald equation. HbA_1C_ was measured using high-performance liquid chromatography (Tosoh G7, Tosoh Corporation, Tokyo, Japan). The coefficient of variation for HbA_1C_ assay ranged from 1.4 to 1.9%. A National Glycohemoglobin Standardization Program-certified assay was used to measure HbA_1C_. Fasting plasma glucose was measured using the glucose oxidase method. Glucose assays were run in duplicate; the intra-assay coefficient of variation was < 3%. Circulating brain natriuretic peptide (BNP) levels were measured by chemiluminescent immunoassay performed on an immunoassay system (ADVIA Centaur; Siemens), with an intra-assay coefficient of variation, 4.2%, 3.1%, and 3.4% for 3 BNP concentrations, respectively [24]. High-sensitivity cardiac troponin-I (hs-cTnI) was measured with the ARCHITECT hs-cTnI assay platform (Abbott Diagnostics), a 2-step, double-monoclonal immunoassay that uses antibody-coated paramagnetic microparticles. The assay has a coefficient of variation of 10% at a concentration of 3.0 ng/L [25].

The main clinical outcome of interest was a composite of major cardiovascular adverse event defined as the first occurrence of any of the following fatal and non-fatal cardiovascular outcomes: coronary artery disease, stroke, heart failure, and atrial fibrillation, between the date of a participant’s first visit and December 31, 2016. The events were identified through a physician-led adjudication process in the Jackson Heart Study, which has been described previously [26]. The identification of incident coronary heart disease (fatal or nonfatal myocardial infarction or coronary revascularization), stroke (fatal and non-fatal ischemic and hemorrhagic stroke), heart failure, and atrial fibrillation was done in a two-step process including the use of the relevant International Classification of Diseases codes from hospital records, followed by adjudication [26].

The initial analytical approach was to create four clinical groups based on the presence or absence of obesity and/or hypertension. These included: (1) patients with isolated diabetes; (2) diabetes and hypertension; (3) diabetes and obesity; and (4) diabetes, obesity, and hypertension. We explored the differential distribution of cardiovascular risk factors (demographics, hemodynamics and biochemical as well as anti-diabetic medications) and the ability of these clinical groups to define the future risk of cardiovascular outcome. We then performed agglomerative hierarchical clustering analysis of individuals based on clinical and biochemical (n = 21) and the echocardiographic (n = 8) variables. Hierarchical clustering naturally produces structures that are informative and thus easy to determine the number of clusters [15]. The algorithm assumes that individuals with closer data points in space, exhibit more similarity to each other than those with data points that are farther away. We used the Ward approach, which starts by classifying all individuals into a single cluster and then partitions as the distance increases, aiming to minimize the within cluster variance. This approach also works well for quantitative variables. To arrive at the optimum number of clusters, we applied a suite of 30 indices in the NbClust package implemented in R [16]. This function uses up to 30 indices to determine the number of clusters and proposes the best clustering scheme from the different results obtained by varying all combinations of number of clusters, distance measures, and clustering methods. In our dataset, we determined that three clusters were the optimum, explaining a total of 74% cumulative variance and with 2.25, 1.27 and 0.93 Eigen values. Through clustering, we grouped subjects with similar overall functional profile to create homogeneous clusters of diabetes patients, and the key differentiating factors being: LV mass (indexed to body surface area), LEDV, LVESV, LVEF; E/A ratio and LA diameter (indexed to body surface area).

We compared the characteristics of participants across the clinical groups (according to presence of obesity and/or hypertension) and the clusters (1, 2 & 3) using ANOVA or Kruskal-Wallis test for continuous variables, and the Chi-square or Fischer exact tests for categorical variables. The comparison of continuous variables were followed by post hoc tests for pairwise comparisons in case of overall significance and applying Bonferroni correction for test multiplicity. Distribution of echocardiographic parameters were further compared across groups using linear regression models adjusting for age and sex. We elected to only adjust for these two variables as clinical variables that would be potential confounders were part of the clusters building.

Survival analyses based on time-to-event data were then performed to assess the prognostic value of the clinical groups and the identified clusters. Crude incidence rates and 95% confidence intervals (CIs) were calculated by exposure levels (clinical groups and clusters). The person-time of follow up from baseline until the first occurrence of (a) cardiovascular disease outcomes, (b) death, or (c) censoring (date of the last available follow-up). The differences between event-free survivor probabilities between the different groups were compared using the log-rank test. For multivariable analysis, we fitted Cox proportional hazards regression models to relate each clinical groups or cluster to incident cardiovascular disease, after verification of the assumption of proportionality of hazards tested using Schoenfeld residuals. The adjustment variables included age and sex, for both the comparison of clusters and the clinical groups. For the clinical groups, the adjustment for these variables already provided with an idea of the significance of the comparisons, thus obviating the need for further adjustment.

Two-sided *P* values of < 0.05 were considered statistically significant, including for interaction terms. All analyses were performed using SAS 9.4 (SAS Institute, Cary, NC) including clustering analyses and visualizations.

## Results

The characteristics of the three clusters are shown in Tables 1 and 2. Cluster 3 was characterized by the highest LV. Cluster 1 had intermediate LVMi, LV volumes, LVEF, LA dimension, and E/A ratio, as well as levels of hsTnI, between clusters 2 & 3. Cluster 2 had the lowest LVMi, lowest LV volumes, highest E/A ratio, and highest LVEF. This cluster comprised predominantly female patients with isolated diabetes. Cluster 3 had the highest LVMi, highest LV volumes, highest LA diameter, lowest E/A ratio, and intermediate LVEF. Cluster 3 gathered the oldest patients with the lowest frequency of isolated diabetes, highest systolic blood pressure, the highest BNP and hsTnI levels.

**Table 1:** Clinical characteristics of participants by phenotypic clusters in the Jackson Heart Study

Dependent	Cluster 1 (n = 260)	Cluster 2 (n = 150)	Cluster 3 (n = 119)	*P *value
Clinical characteristics				
Age, years	59.20 ± 0.64	60.58 ± 0.85	62.71 ± 0.953^‡^	0.0003
Women, n (%)	188 (72.3)	105(70.0)	67 (56.3)^‡^	0.0068
Body mass index, kg/m^2^	35.68 ± 0.44	33.96 ± 0.58	32.48 ± 0.66^†‡^	0.0002
Obesity, n (%)	193 (74.2)	102 (68.0)	72 (60.5)^‡^	0.016
Current smokers, n (%)	21 (8.1)	20 (13.3)	13 (10.9)	0.23
Hypertension, n (%)	197 (75.8)	116 (77.3)	101 (84.9)	0.12
Group, n (%)				
Isolated T2DM	11 (27.5)	19 (47.5)	10 (25.0)	0.037
T2DM + Obesity	32 (43.8)	33 (45.2)	10 (11.0)	
T2DM + HTN	48 (40.0)	38 (31.8)	34 (28.3)	
T2DM + Obesity + HTN	119 (41.2)	118 (40.8)	55 (18.0)	
Hemodynamics				
Heart rate, bpm	69.35 ± 0.68	66.36 ± 0.89^†^	67.18 ± 1.00^‡^	0.018
Systolic BP, mmHg	128.30 ± 1.10	129.63 ± 1.34^†^	137.34 ± 1.50^‡^	< 0.0001
Diastolic BP, mmHg	74.61 ± 0.52	73.87 ± 0.69	75.02 ± 0.77	0.51
Biochemistry				
HbA_1C_, %	7.67 ± 0.09	7.41 ± 0.12	7.27 ± 0.14	0.048
HDL-cholesterol, mg/dL	49.80 ± 0.85	50.38 ± 1.12	51.02 ± 1.26	0.72
LDL-cholesterol, mg/dL	126.76 ± 2.28	117.42 ± 3.00	123.20 ± 3.37	0.048
Triglycerides, mg/dL	124.05 ± 3.84	120.05 ± 5.06	114.75 ± 5.68	0.39
eGFR, mL/min/1.73 m^2^	94.51 ± 1.44	91.64 ± 1.89	87.51 ± 2.12	0.019
BNP, pg/dL	11.86 ± 1.82	17.68 ± 2.40^†^	25.55 ± 2.70^‡^	0.0001
High sensitivity troponin I, pg/dL	5.58 ± 1.64	8.91 ± 2.15	14.30 ± 2.42*	0.012
Medications, n (%)				
Metformin	42 (16.2)	25 (16.7)	18 (15.1)	0.94
Insulin	36 (13.8)	15 (10.0)	19 (16.0)	0.33
ACE inhibitors	29 (11.2)	17 (11.3)	10 (8.4)	0.67
Other diabetes medications	72 (27.7)	54 (36.0)	42 (35.3)	0.14
Statins therapy	119 (46.8)	71 (48.0)	58 (50.0)	0.85

^†^p < 0.05 compared with cluster 1; ^‡^p < 0.05 compared with cluster 2; Bonferroni corrected p-value = 0.05/4 = 0.0125

Values are reported as mean ± SD for continuous traits and n (%) for dichotomous traits. ACE: angiotensin converting enzyme, BNP: brain natriuretic peptide, BP: blood pressure, eGFR: estimated glomerular filtration rate, HDL: high-density lipoprotein, HTN: hypertension, LDL: low-density lipoprotein, T2DM: type 2 diabetes mellitus

**Table 2:** Echocardiographic characteristics of the various identified clusters in the Jackson Heart Study

Echocardiographic characteristics	Cluster1 (n = 260)	Cluster 2 (n = 150)	Cluster 3 (n = 119)	*P * value
LVMI (g/m^2^)	64.23 ± 0.71	77.29 ± 0.94	104.92 ± 1.05	< 0.0001
LVEDV (mL)	142.44 ± 1.56	170.28 ± 2.05	182.23 ± 2.30	< 0.0001
LVEDVi (mL/m^2^)	68.29 ± 0.67	84.09 ± 0.89	91.33 ± 1.00	< 0.0001
LVESV (mL)	50.37 ± 1.08	59.46 ± 1.42	68.80 ± 1.60	< 0.0001
LVESVi (mL/m^2^)	24.08 ± 0.49	29.27 ± 0.65	34.41 ± 0.73	< 0.0001
LVEF (%)	63.02 ± 0.40	64.03 ± 0.52	63.11 ± 0.59^ns^	0.28
LAi (mm/m^2^)	16.8 ± 0.20	17.9 ± 0.20	18.3 ± 0.20	< 0.0001
E/A ratio	0.98 ± 0.02	0.97 ± 0.02^ns^	0.91 ± 0.02	0.053

Values are reported as mean ± SD—All comparisons with mean in cluster 1 were significant

E/A: ratio between peak early and late diastolic velocities; LAi: left atrial diameter indexed; LVEDV: left ventricular end-diastolic volume; LVEDVi : left ventricular end-diastolic volume indexed to body surface area; LVEF: left ventricular ejection fraction; LVESV = left ventricular end-systolic volume; LVESVi : left ventricular end-systolic volume mass indexed to body surface area; LVMi = left ventricular mass indexed to body surface area

The characteristics of the participants by clinical groups only (diabetes only, diabetes and obesity, diabetes and hypertension and diabetes and obesity and hypertension) are summarized in Additional file 1: Table S2. Participants in the diabetes, and obesity and hypertension group were more likely to be women, have an elevated heart rate, high systolic blood pressure, low estimated glomerular filtration rate, and to be on angiotensin converting enzyme inhibitors or on statins. They were less likely to be smokers. Additional file 1: Table S3 shows the comparisons of echocardiographic data among the four clinical groups. The differences in LV morphology (LV mass index and LV volumes) observed in unadjusted analyses, did not persist in adjusted analyses (accounting for age and sex). Obesity was associated with an abnormal diastolic function, with groups with obesity having a higher E/A ratio, compared with the other groups. Overall, there was an important overlap of individual values of systolic parameters among the four groups.

**Fig. 1:**
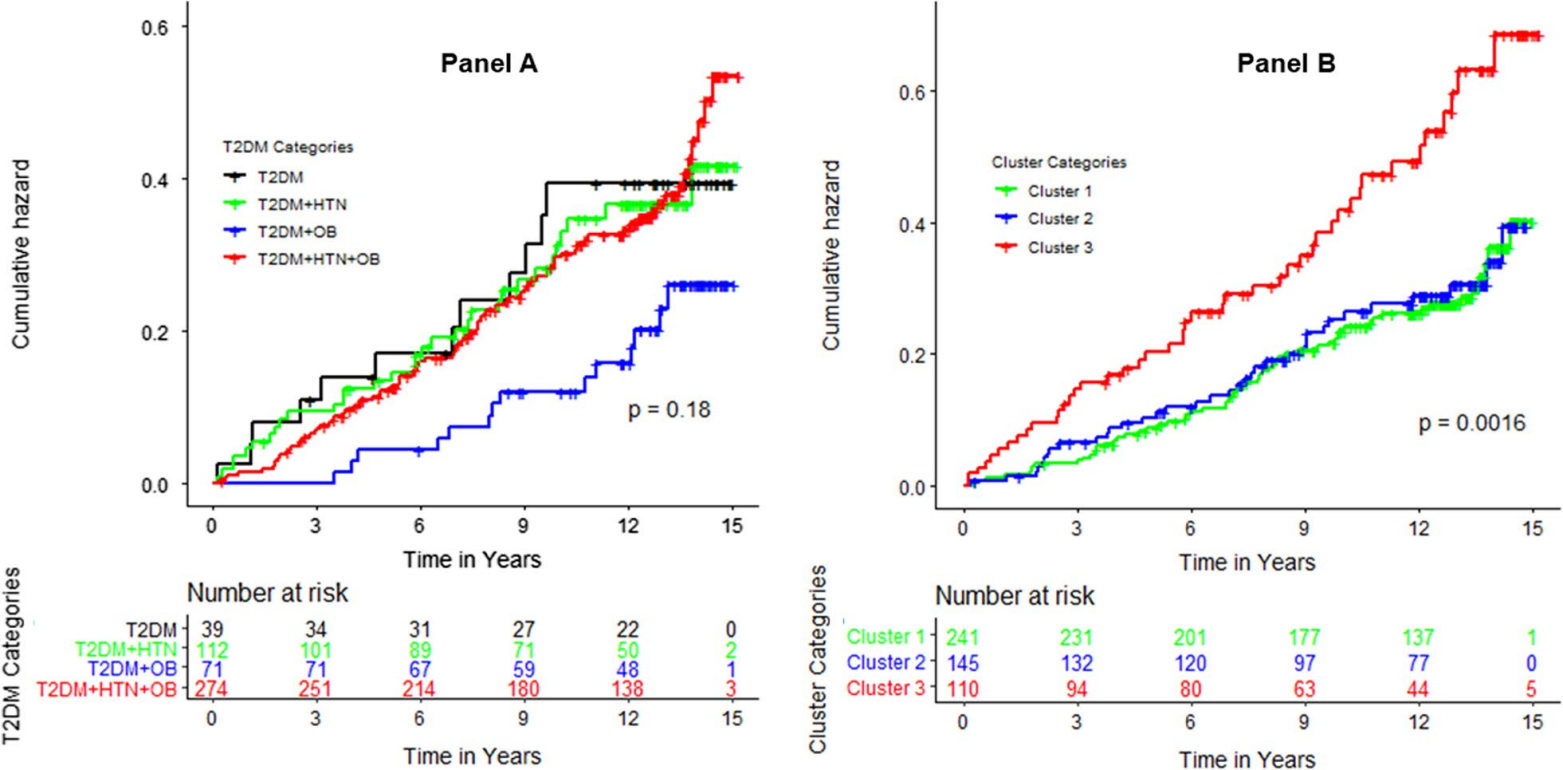
Incidence of cardiovascular events by Clinical Categories and by Clusters. **A** Clinical categories, **B** clusters. HTN: hypertension, OB: obesity T2DM: type 2 diabetes mellitus

Among 529 Blacks with diabetes (68% women, mean age 60 ± 10 years), 141 incident cardiovascular events (41 coronary heart disease, 36 stroke, 43 heart failure and 21 atrial fibrillation events) observed over a median follow-up of 12 years (range 1 to 15 years). The overall incidence rate of cardiovascular disease in our sample was 26.3 (95% CI 22.4, 31.0) per 1000 person-years. The incidence rate of cardiovascular disease by clinically relevant categories and clusters is shown in Fig. 1; Table 3.

**Table 3:** Incidence of cardiovascular events by clinical groups or phenotypic clusters

	Event rates per 1000 person-years (95% CI)	Adjusted hazard ratio (95% CI)^‡^
Clusters		
Cluster 1	22.95 (17.82, 29.55)	Reference
Cluster 2	23.86 (17.21, 33.08)	1.11 (0.73, 1.68)
Cluster 3	43.93 (32.80, 58.83)	1.60 (1.08, 2.37)
Clinical categories		
Isolated T2DM	29.75 (16.90, 52.39)	Reference
T2DM and obesity	16.32 (9.66, 27.55)	0.74 (0.33, 1.63)
T2DM and HTN	29.22 (20.67, 41.32)	0.84 (0.42, 1.65)
T2DM, HTN and obesity	29.73 (23.98, 36.87)	1.05 (0.56, 1.96)

^‡^Age- and sex-adjusted hazard ratio; CI: confidence interval, T2DM: Type 2 diabetes mellitus, HTN: Hypertension

In multivariable adjusted Cox proportional hazards models (Table 3), Cluster 3 had a worse prognostic value, in terms of incident cardiovascular disease, than Cluster 1 (hazard ratio: 1.60; 95% CI 1.08 to 2.37); the prognostic value of Cluster 2 was not significantly different from that of Cluster 1 (hazard ratio: 1.11; 95% CI 0.73 to 1.68). On the other hand, compared to individuals in the isolated diabetes, those in the diabetes and hypertension group (hazard ratio: 0.74, 95% CI: 0.33 to 1.63), or diabetes and obesity group (hazard ratio: 0.84; 95% CI 0.42–1.65), or diabetes and hypertension, and obesity group (hazard ratio: 1.05; 95% CI 0.56 to 1.96) groups, were not at higher risk of cardiovascular events.

## Discussion

In a community-based sample of Blacks with diabetes, we assessed the risk for cardiovascular events, based on clinical and echocardiographic data, and an innovative statistical approach (cluster analysis). We made a number of observations. First, whereas classical statistical analysis, based on a priori risk factor groups, resulted in a substantial overlap of the groups, cluster analysis was able to distinguish three groups mainly differentiated by echocardiographic indices and cardiac biomarkers. Clinical characteristics varied between these clusters, with phenotypes associated not only with obesity and hypertension but also with age and sex. Second, the cluster of participants with the worse alteration in both left ventricular structure-function and the highest levels of markers of myocardial stress (BNP) and injury (hsTnI) had the worse cardiovascular prognosis.

Diabetes is a heterogeneous condition, given its coexistence with hypertension and obesity. The latter conditions make it difficult to isolate the intrinsic contribution of glucose dysregulation to myocardial dysfunction in human studies, as these comorbid conditions can also affect cardiac remodeling. Using an a priori hypothesis based on the comorbidities associated with diabetes, namely obesity and hypertension, this afforded limited discrimination in terms of future risk of cardiovascular disease. The cluster analyses showed three phenotypes (mainly based on the echocardiographic indices and cardiac biomarkers): a hypertrophic high-risk phenotype (Cluster 3), and two other Clusters 1 & 2. Cluster 3 had the highest predictive values in terms of incident cardiovascular disease. Thus, our cluster analysis highlighted the prognostic value of LV remodeling and subclinical LV dysfunction in diabetes, despite similar clinical profiles of obesity and hypertension. This suggests that diabetes patients with decreasing LVEF and/or increased LV mass, as well as high levels of biomarkers of cardiac stress and/or injury (BNP and hsTnI) might be suitable for targeted preventive strategies. Furthermore, that BNP and hsTnI were highest in the phenotype (cluster 3) that has the highest prognostic value is not surprising, as these two biomarkers are both representative of subclinical myocardial stress and injury, respectively. BNP has been shown to have a prognostic value among individuals without overt cardiovascular disease [24]. Similarly, subclinical myocardial injury, as assessed by high sensitivity troponin, has also been shown to predict adverse cardiovascular events [27].

Our observations provide additional insights into the relation of diabetes and cardiovascular outcomes, and highlights the key prognostic value of myocardial alterations, in the absence or presence of comorbidities, as well as in the absence of overt cardiovascular disease. The majority of prior studies in the setting of diabetes have seldom evaluated both clinical and echocardiographic parameters in terms of prediction of cardiovascular disease [28–32]. Our findings are consistent with the few previous reports on the prognostic values of subclinical myocardial changes among individuals with diabetes, including LV systolic dysfunction [33, 34], diastolic dysfunction [35]. A prior study has used a cluster analysis approach, and described the prognosis importance of echocardiographic measures among diabetic patients [36]. Our observations expands the latter study, which did not include black participants ( who are disproportionaly affected by diabetes and cardiovascular diseases in the United States [1, 2]), or biomarkers of ventricular wall stress (BNP) or myocardial injury (hsTnI) [36].

The predominance of echocardiographic variables in the clusters most probably illustrates the various mechanistic processes leading to cardiac remodeling in diabetes [37]. On one hand, diabetes increases cardiomyocyte hypertrophy and stiffness, because of hyperinsulinemia, microvascular endothelial inflammation and microvascular rarefaction [37, 38], leading to a phenotype with preserved ejection fraction. On the other hand, diabetes augments fibrosis because of cardiomyocyte death induced by lipotoxicity and/or advanced glycation end products [37, 38], leading to a reduction in ejection fraction.

Our study suggests that among Blacks with diabetes, structural myocardial dysfunction and cardiac biomarkers are potentially key determinants of cardiovascular prognosis. Thus, our findings points to the potential utility of cluster analysis to risk stratify, and this select individuals without overt heart failure or cardiovascular disease in general who may benefit from novel diabetes therapies with cardiovascular benefits, namely the SGLT-2 inhibitors [7], and the GLP-1 receptors agonists [8], which are now recommended in guidelines for use the context of diabetes, to optimize cardiovascular protection [39].

The strengths of this study include a well-characterized community-based sample of Blacks, the availability of both clinical, echocardiographic, and cardiac biomarkers data, and the use of an innovative analytical approach to analysis to identify clusters of patients with unique phenotypes with a prognosis value. Indeed, contrary to classic statistical analysis, cluster analysis is a machine learning and exploratory technique that provides tools to identify unknown subgroups but with distinct characteristics that carry a prognosis values [15, 16].

Some limitations of our study should be acknowledged. First, our analysis lacked power to investigate the individual cardiovascular disease events (coronary heart disease, stroke, heart failure and atrial fibrillation), thus we used a composite endpoint. Second, the participants were Blacks in Jackson, Mississippi; thus, results may not be generalizable to other ethnic groups or Blacks elsewhere in the United States. Third, we did not include all the potentially relevant echocardiographic indices (relating to systolic and diastolic functions) such as LA volumes, tissue Doppler measures, and strain measures, which may have helped to refine the definition of the clusters. Fifth, we did not have data on key diabetes-related factor such as the disease duration and microvascular complications (such as retinopathy [40, 41], autonomic neuropathy [42], or erectile dysfunction [43], shown to be related to myocardial alterations and cardiovascular outcomes), which can help in refining the assessment of the risk of cardiovascular disease in the context of diabetes. Fourth, we focused on select group of diabetic participants with complete data on the various variables, which likely introduced some selection bias, but this is an inevitable phenomenon in observational studies. Lastly, because of the observational nature of our analysis, the study findings may be predisposed to residual confounding.

In conclusion, in a community-based sample of black adults, cluster identification revealed three phenotypes among patients with diabetes, indicating that despite similar clinical profiles, patients with a phenotype characterized by the highest LVMI, highest LV volumes, lowest LVEF, lowest E/A ratio, and elevated cardiac biomarkers (BNP and hsTnI) are at higher cardiovascular risk. These findings underscore the importance of detecting of subtle myocardial abnormalities and elevation in cardiac biomarkers, which can help in reliability predicting future cardiovascular risk among individuals with diabetes.

.

## Supplementary Information

**Additional file 1. **Supplementary study material (Tables and Figure).

## Data Availability

The data is available upon request from the author and conditional on obtaining the relevant authorization from the Jackson heart Study.

## References

[1] Virani SS, Alonso A, Aparicio HJ, Benjamin EJ, Bittencourt MS, Callaway CW (2021). Heart disease and stroke statistics—2021 update: a report from the American Heart Association. Circulation..

[2] Menke A, Casagrande S, Geiss L, Cowie CC (2015). Prevalence of and trends in diabetes among adults in the United States, 1988–2012. JAMA.

[3] Peters SAE, Huxley RR, Woodward M (2014). Diabetes as risk factor for incident coronary heart disease in women compared with men: a systematic review and meta-analysis of 64 cohorts including 858,507 individuals and 28,203 coronary events. Diabetologia.

[4] Peters SAE, Huxley RR, Woodward M (2014). Diabetes as a risk factor for stroke in women compared with men: a systematic review and meta-analysis of 64 cohorts, including 775 385 individuals and 12 539 strokes. Lancet.

[5] Ohkuma T, Komorita Y, Peters SAE, Woodward M (2019). Diabetes as a risk factor for heart failure in women and men: a systematic review and meta-analysis of 47 cohorts including 12 million individuals. Diabetologia..

[6] Huxley RR, Filion KB, Konety S, Alonso A (2011). Meta-analysis of cohort and case-control studies of type 2 diabetes mellitus and risk of atrial fibrillation. Am J Cardiol.

[7] McGuire DK, Shih WJ, Cosentino F, Charbonnel B, Cherney DZI, Dagogo-Jack S (2021). Association of SGLT2 inhibitors with cardiovascular and kidney outcomes in patients with type 2 diabetes: a meta-analysis. JAMA Cardiol.

[8] Kristensen SL, Rørth R, Jhund PS, Docherty KF, Sattar N, Preiss D (2019). Cardiovascular, mortality, and kidney outcomes with GLP-1 receptor agonists in patients with type 2 diabetes: a systematic review and meta-analysis of cardiovascular outcome trials. Lancet Diabetes Endocrinol..

[9] Hiruma S, Shigiyama F, Hisatake S, Mizumura S, Shiraga N, Hori M (2021). A prospective randomized study comparing effects of empagliflozin to sitagliptin on cardiac fat accumulation, cardiac function, and cardiac metabolism in patients with early-stage type 2 diabetes: the ASSET study. Cardiovasc Diabetol l.

[10] Katakami N, Mita T, Yoshii H, Shiraiwa T, Yasuda T, Okada Y (2021). Effect of tofogliflozin on arterial stiffness in patients with type 2 diabetes: prespecified sub-analysis of the prospective, randomized, open-label, parallel-group comparative UTOPIA trial. Cardiovasc Diabetol.

[11] Colosia AD, Palencia R, Khan S (2013). Prevalence of hypertension and obesity in patients with type 2 diabetes mellitus in observational studies: a systematic literature review. Diabetes Metab Syndr Obes Targets Ther.

[12] Wong CY, O’Moore-Sullivan T, Leano R, Byrne N, Beller E, Marwick TH (2004). Alterations of left ventricular myocardial characteristics associated with obesity. Circulation.

[13] Ho JE, McCabe EL, Wang TJ, Larson MG, Levy D, Tsao C (2017). Cardiometabolic traits and systolic mechanics in the community. Circ Heart Fail.

[14] Cheng S, Xanthakis V, Sullivan LM, Lieb W, Massaro J, Aragam J (2010). Correlates of echocardiographic indices of cardiac remodeling over the adult life course: longitudinal observations from the Framingham heart study. Circulation.

[15] Eshghi A, Haughton D, Legrand P, Skaletsky M, Woolford S (2011). Identifying groups: a comparison of methodologies. J Data Sci.

[16] Charrad M, Ghazzali N, Boiteau V, Niknafs A, NbClust (2014). An R package for determining the relevant number of clusters in a data set. J Stat Softw.

[17] Taylor HA (2005). The Jackson Heart Study: an overview. Ethn Dis.

[18] American Diabetes Association (2019). 2. Classification and diagnosis of diabetes: standards of medical care in diabetes 2019. Diabetes Care..

[19] Devereux RB, Alonso DR, Lutas EM, Gottlieb GJ, Campo E, Sachs I (1986). Echocardiographic assessment of left ventricular hypertrophy: comparison to necropsy findings. Am J Cardiol.

[20] de Simone G, Devereux RB, Daniels SR, Koren MJ, Meyer RA, Laragh JH (1995). Effect of growth on variability of left ventricular mass: assessment of allometric signals in adults and children and their capacity to predict cardiovascular risk. J Am Coll Cardiol.

[21] Nagueh SF, Smiseth OA, Appleton CP, Byrd BF, Dokainish H, Edvardsen T (2016). Recommendations for the evaluation of left ventricular diastolic function by echocardiography: an update from the American society of echocardiography and the European Association of Cardiovascular Imaging. J Am Soc Echocardiogr.

[22] Levey AS, Stevens LA, Schmid CH, Zhang YL, Castro AF, Feldman HI (2009). A new equation to estimate glomerular filtration rate. Ann Intern Med.

[23] Carpenter MA, Crow R, Steffes M, Rock W, Heilbraun J, Evans G (2004). Laboratory, reading center, and coordinating center data management methods in the Jackson Heart Study. Am J Med Sci.

[24] Fox ER, Samdarshi TE, Musani SK, Pencina MJ, Sung JH, Bertoni AG (2016). Development and validation of risk prediction models for cardiovascular events in black adults: the Jackson heart study cohort. JAMA Cardiol.

[25] Apple FS, Collinson PO (2012). Analytical characteristics of high-sensitivity cardiac troponin assays. Clin. Chem.

[26] Keku E, Rosamond W, Taylor HA, Garrison R, Wyatt SB, Richard M (2005). Cardiovascular disease event classification in the Jackson Heart Study: methods and procedures. Ethn Dis.

[27] McEvoy JW, Chen Y, Ndumele CE, Solomon SD, Nambi V, Ballantyne CM (2016). Six-year change in high-sensitivity cardiac troponin T and risk of subsequent coronary heart disease, heart failure, and death. JAMA Cardiol.

[28] Devereux RB, Roman MJ, Paranicas M, O’Grady MJ, Lee ET, Welty TK (2000). Impact of diabetes on cardiac structure and function: the Strong Heart Study. Circulation.

[29] Liu JE, Palmieri V, Roman MJ, Bella JN, Fabsitz R, Howard BV (2001). The impact of diabetes on left ventricular filling pattern in normotensive and hypertensive adults: the strong heart study. J Am Coll Cardiol.

[30] Rutter MK, Parise H, Benjamin EJ, Levy D, Larson MG, Meigs JB (2003). Impact of glucose intolerance and insulin resistance on cardiac structure and function: sex-related differences in the Framingham Heart Study. Circulation.

[31] Skali H, Shah A, Gupta DK, Cheng S, Claggett B, Liu J (2015). Cardiac structure and function across the glycemic spectrum in elderly men and women free of prevalent heart disease: the atherosclerosis risk in the community study. Circ Heart Fail.

[32] Bertoni AG, Goff DC, D’Agostino RB, Liu K, Hundley WG, Lima JA (2006). Diabetic cardiomyopathy and subclinical cardiovascular disease. Diabetes Care.

[33] Holland DJ, Marwick TH, Haluska BA, Leano R, Hordern MD, Hare JL (2015). Subclinical LV dysfunction and 10-year outcomes in type 2 diabetes mellitus. Heart.

[34] Rørth R, Jhund PS, Mogensen UM, Kristensen SL, Petrie MC, Køber L (2018). Risk of incident heart failure in patients with diabetes and asymptomatic left ventricular systolic dysfunction. Diabetes Care.

[35] From AM, Scott CG, Chen HH (2010). The development of heart failure in patients with diabetes mellitus and pre-clinical diastolic dysfunction. A population-based study. J Am Coll Cardiol.

[36] Ernande L, Audureau E, Jellis CL, Bergerot C, Henegar C, Sawaki D (2017). Clinical implications of echocardiographic phenotypes of patients with diabetes mellitus. J Am Coll Cardiol.

[37] van Heerebeek L, Hamdani N, Handoko ML, Falcao-Pires I, Musters RJ, Kupreishvili K (2008). Diastolic stiffness of the failing diabetic heart: importance of fibrosis, advanced glycation end products, and myocyte resting tension. Circulation.

[38] Paulus WJ, Dal Canto E (2018). Distinct myocardial targets for diabetes therapy in heart failure with preserved or reduced ejection fraction. JACC Heart Fail.

[39] Das SR, Everett BM, Birtcher KK, Brown JM, Cefalu WT, Januzzi JL (2020). 2020 ACC Expert Consensus decision pathway on novel therapies for cardiovascular risk reduction in patients with type 2 diabetes and atherosclerotic cardiovascular disease. J Am Coll Cardiol.

[40] Cheung N, Jie JW, Klein R, Couper DJ, Sharrett AR, Wong TY (2007). Diabetic retinopathy and the risk of coronary heart disease: the atherosclerosis risk in communities study. Diabetes Care.

[41] Cheung N, Wang JJ, Rogers SL, Brancati F, Klein R, Sharrett AR (2008). Diabetic retinopathy and risk of heart failure. J Am Coll Cardiol.

[42] Sacre JW, Franjic B, Jellis CL, Jenkins C, Coombes JS, Marwick TH (2010). Association of cardiac autonomic neuropathy with subclinical myocardial dysfunction in type 2 diabetes. JACC Cardiovasc Imaging.

[43] Yamada T, Hara K, Umematsu H, Suzuki R, Kadowaki T (2012). Erectile dysfunction and cardiovascular events in diabetic men: a meta-analysis of observational studies. PLoS ONE.

