# Supplementary material for: Patients phenotypes and cardiovascular risk in type 2 diabetes: the Jackson Heart Study
Source: Cardiovasc Diabetol. 2022 Jun 1;21:89. doi: 10.1186/s12933-022-01501-z (PMC9161484; doi:10.1186/s12933-022-01501-z)
Supplement: Supplementary file 1 — Additional file 1. Supplementary study material (Tables and Figure). [file 12933_2022_1501_MOESM1_ESM.docx]

**SUPPLEMENTAL DATA**

**Table S1**: Comparison between included and excluded participants to the Jackson Heart Study Exam 1 (2000-2004)

|  | **Included (n=529)** | **Excluded (n=594)** | p-Value |
| --- | --- | --- | --- |
| Age, years | 60±11 | 60±10 | 0.80 |
| Women, % | 354 (67.0) | 265 (44.6) | 0.89 |
| Body Mass Index, kg/m^2^ | 34.5±7.3 | 33.9±7.1 | 0.26 |
| Obesity, N (%) | 367 (69.4) | 260 (68.1) | 0.67 |
| Current Smokers, % | 54 (10.2) | 38 (6.4) | 0.09 |
| Hypertension, % | 414 (78.3) | 313 (52.7) | 0.02 |
| **Hemodynamics** |  |  |  |
| Heart rate, beats/s | 68.0±11.0 | 69.1±12.0 | 0.18 |
| Systolic BP, mmHg | 130.7±16.7 | 131.3±16.7 | 0.59 |
| Diastolic BP, mmHg | 74.5±8.4 | 74.0±8.7 | 0.41 |
| **Biochemistry** |  |  |  |
| HbA_1C_ , % | 7.50±1.50 | 7.82±1.98 | 0.009 |
| HDL-cholesterol, mg/dL | 50.2±13.7 | 49.6±13.3 | 0.56 |
| LDL-cholesterol, mg/dL | 123.3±36.9 | 128.1±38.1 | 0.13 |
| Triglycerides, mg/dL‡ | 105.0 (77.0, 151.0) | 110.5 (78.0, 173.0) | 0.086 |
| eGFR, ml/min/1.73 m^2^ | 92.1±23.3 | 88.6±25.4 | **0.033** |
| BNP, pg/dL‡ | 7.00 (2.73, 17.82) | 10.72 (3.30, 25.72) | 0.013 |
| hsTroponin, pg/dL‡ | 3.41 (2.43, 6.24) | 3.69 (2.71, 6.09) | 0.13 |
| **Medications, n (%)** |  |  |  |
| Metformin | 85 (16.1) | 70 (11.8) | 0.034 |
| Insulin | 70 (13.2) | 89 (15.0) | <0.001 |
| ACE inhibitors | 56 (10.6) | 26 (4.4) | 0.028 |
| Other diabetes medications | 168 (31.6) | 123 (20.7) | 0.040 |
| Statins Therapy | 140 (26.5) | 97 (16.3) | 0.050 |

Means ± Standard Deviation for continuous variables, comparison done using 2-sample T-test; ‡Median (25^th^, 75^th^ percentiles) comparison done using Wilcoxon 2-sample test; chi-square test is used to test association between categorical variables.

**Table S2: Clinical and Biological Characteristics of Jackson Heart Study participants**

| **Clinical characteristics** | **Isolated T2DM**  **(n=40)** | **T2DM + Obesity**  **(n=75)** | **T2DM + HTN (n=122)** | **T2DM + HTN + Obesity (n=292)** | ***P Value*** |
| --- | --- | --- | --- | --- | --- |
| Age, years | 59.32±1.56 | 52.51±1.14* | 64.98±0.90*† | 59.74±0.58†‡ | <0.0001 |
| Women, n (%) | 13 (32.5) | 53 (68.9)* | 72 (59.0)† | 222 (76.0)*‡ | <0.0001 |
| Body mass index, kg/m^2^ | 26.80±0.70 | 37.20±0.51* | 27.05±0.38† | 37.43±0.25*‡ | <0.0001 |
| Obesity, n (%) | - | 75 (100.0) | - | 292 (100.0) | - |
| Current smokers, n (%) | 10 (25.0) | 11 (14.7) | 10 (8.2)* | 23 (7.9)* | 0.0006 |
| Hypertension, n (%) | - | - | 122 (100.00) | 292 (100.0) | - |
| **Hemodynamics** |  |  |  |  |  |
| Heart rate, beats/min | 66.05±1.7345 | 70.00±1.04 | 64.58±0.78† | 68.99±0.51‡ | 0.0004 |
| Systolic BP, mmHg | 122.41±2.11 | 121.77±1.52 | 134.39±1.14*† | 132.63±0.75*† | <0.0001 |
| Diastolic BP, mmHg | 72.24±1.11 | 73.06±0.80 | 74.21±0.60 | 74.67±0.39 | 0.087 |
| **Biochemistry** |  |  |  |  |  |
| HbA_1C_ , % | 7.75±0.20 | 7.62±0.15 | 7.5725±0.11 | 7.57±0.087 | 0.044 |
| HDL-cholesterol, mg/dL | 49.94±1.96 | 48.68±1.39 | 51.72±1.12 | 50.04±0.71 | 0.375 |
| LDL-cholesterol, mg/dL | 122.71±5.30 | 127.61±3.75 | 124.33±3.03 | 123.17±1.93 | 0.757 |
| Triglycerides, mg/dL | 113.47±15.42 | 127.90±10.96 | 123.08±8.78 | 138.23±5.69 | 0.281 |
| eGFR, ml/min/1.73 m^2^ | 95.29±3.04 | 103.96±2.20 | 83.91±1.65*† | 90.08±1.09† | <.0001 |
| BNP, pg/dL | 13.01±4.34 | 7.73±3.17 | 24.88±2.40† | 18.01±1.55 | 0.0002 |
| hsTroponin, pg/dL | 4.09±3.36 | 3.74±2.34 | 9.53±1.77 | 9.82±1.16 | 0.058 |
| **Medications, n (%)** |  |  |  |  |  |
| Metformin | 9 (22.5) | 9 (12.0) | 16 (13.1) | 51 (17.5) | 0.83 |
| Insulin | 2 (5.0) | 5 (6.7) | 17 (13.9) | 46 (15.8) | 0.14 |
| ACE inhibitors | 1 (2.5) | 0 (0.0) | 17 (13.9)† | 38 (13.0)^†^ | <0.0001 |
| Other diabetes medications | 8 (20.0) | 18 (24.0) | 44 (36.1) | 98 (33.6) | 0.0036 |
| Statins Therapy | 16 (39) | 57 (77) | 18 (14) | 134 (43)**^†^** | 0.0015 |

**Key^+^**: *p<0.005 compared with T2DM; †p<0.05 compared with T2DM + Obesity; ‡p<0.05 compared with T2DM + HTN. **^+^** = Bonferroni correction (p<0.05 for 6 tests = 0.0083). Values are reported as mean ± SD for continuous traits and n (%) for dichotomous traits. ACE: angiotensin converting enzyme, BNP: brain natriuretic peptide, BP: blood pressure, CHD: coronary heart disease, eGFR: estimated glomerular filtration rate, HDL: high-density lipoprotein, HTN: hypertension, LDL: low-density lipoprotein, T2DM: type 2 diabetes

**Table S3: Echocardiographic Characteristics of Jackson Heart Study participants**

| **Predictors** | Isolated T2DM  (n=40) | T2DM + Obesity  (n=75) | T2DM + HTN (n=122) | T2DM + Obesity + HTN  (n=292) | *P Value* |
| --- | --- | --- | --- | --- | --- |
| LVMI (g/m^2^) |  |  |  |  |  |
| Crude | 75.92 ± 3.07 | 70.95 ± 2.24 | 82.16 ± 1.72† | 76.70 ± 1.13‡ | **0.0012** |
| Adjusted | 74.85 ± 3.01 | 74.93 ± 2.30 | 81.09 ± 1.77 | 78.62 ± 1.21 | 0.12 |
| LVEDV (ml) |  |  |  |  |  |
| Crude | 157.96 ± 4.78 | 160.94 ± 3.49 | 153.11 ± 2.74 | 161.62 ± 1.76 | 0.0693 |
| Adjusted | 154.58 ± 4.61 | 164.07 ± 3.54 | 155.37 ± 2.71 | 166.54 ± 1.84 | 0.0022 |
| LVEDVi (ml/m^2^) |  |  |  |  |  |
| Crude | 81.02 ± 2.30 | 75.19 ± 1.68 | 81.19 ± 1.31 | 76.89 ± 0.85 | **0.0074** |
| Adjusted | 80.88 ± 2.31 | 76.24 ± 1.77 | 80.73 ± 1.36 | 77.21 ± 0.92 | 0.08 |
| LVESV (ml) |  |  |  |  |  |
| Crude | 55.95 ± 2.99 | 58.82 ± 2.18 | 53.82 ± 1.71 | 57.90 ± 1.11 | 0.18 |
| Adjusted | 57.75 ± 2.87 | 60.04 ± 2.20 | 55.76 ± 1.69 | 61.00 ± 1.15 | 0.04 |
| LVESVi (ml/m^2^) |  |  |  |  |  |
| Crude | 29.72 ± 1.42 | 27.44 ± 1.04 | 28.48 ± 0.81 | 27.49 ± 0.53 | 0.40 |
| Adjusted | 29.20 ± 1.41 | 27.89 ± 1.08 | 28.83 ± 0.83 | 28.24 ± 0.57 | 0.84 |
| LVEF (%) |  |  |  |  |  |
| Crude | 63.62 ± 1.01 | 61.87 ± 0.74 | 63.89 ± 0.58 | 63.42 ± 0.37 | 0.17 |
| Adjusted | 64.05 ± 0.99 | 62.23 ± 0.76 | 63.15 ± 0.58 | 62.89 ± 0.40 | 0.53 |
| LA Size (mm) |  |  |  |  |  |
| Crude | 33.03±0.67 | 35.32±0.48 | 34.82±0.38 | 36.22±0.25* | **<0.0001** |
| Adjusted | 32.68±0.65 | 35.90±0.50* | 34.88±0.38 | 36.75±0.26*‡ | **<0.0001** |
| LAi (mm//m^2^) |  |  |  |  |  |
| Crude | 16.94±0.37 | 16.55±0.27 | 18.51±0.21* | 17.27±0.14*‡ | **<0.0001** |
| Adjusted | 17.10±0.36 | 16.73±0.28 | 18.21±0.21 | 17.08±0.14‡ | **<0.0001** |
| E/A ratio |  |  |  |  |  |
| Crude | 0.97 ± 0.04 | 0.81.084 ± 0.03 | 0.90 ± 0.03 | 0.96 ± 0.02 | **0.0001** |
| Adjusted | 0.96 ± 0.04 | 1.01 ± 0.03 | 0.95 ± 0.02 | 0.96 ± 0.01† | 0.52 |

**Key**: ‡p<0.0083 compared with DM + HTN; Adjustment are made for age and sex - Values are reported as mean ± SD.

E/A: ratio between peak early and late diastolic velocities; LA: left atrium; LVEDV: left ventricular end-diastolic volume; LVEDVi : left ventricular end-diastolic volume indexed to body surface area; LVEF: [left ventricular ejection fraction](https://www.sciencedirect.com/topics/medicine-and-dentistry/heart-left-ventricle-ejection-fraction); LVESV = left ventricular end-systolic volume; LVESVi : left ventricular end-systolic volume mass indexed to body surface area; LVMi = left ventricular mass indexed to body surface area, HTN: hypertension, LDL: low-density lipoprotein, T2DM: type 2 diabetes mellitus

Jackson Heart Study

Exam 1- n = 5,306

Individuals with Type 2 Diabetes Mellitus = 1,123

Eligible Sample

N = 529

- Prior cardiovascular disease (including history of coronary heart disease, or history of cardiomyopathy/ heart failure including left ventricular regional motion abnormalities/valvular diseases, n=211)
- Missing covariates, including data on echocardiography, (n=26 ) and other covariates (n=326)

Excluded Sample

N=594

**Figure S1**: Flowchart showing exclusions of ineligible participants to exam 1 (2000-2004) of Jackson Heart Study
